# Parallel Aspects of the Microenvironment in Cancer and Autoimmune Disease

**DOI:** 10.1155/2016/4375120

**Published:** 2016-02-22

**Authors:** Michal A. Rahat, Jivan Shakya

**Affiliations:** Immunology Research Laboratory, Carmel Medical Center, and Ruth and Bruce Rappaport Faculty of Medicine, Technion, 3436212 Haifa, Israel

## Abstract

Cancer and autoimmune diseases are fundamentally different pathological conditions. In cancer, the immune response is suppressed and unable to eradicate the transformed self-cells, while in autoimmune diseases it is hyperactivated against a self-antigen, leading to tissue injury. Yet, mechanistically, similarities in the triggering of the immune responses can be observed. In this review, we highlight some parallel aspects of the microenvironment in cancer and autoimmune diseases, especially hypoxia, and the role of macrophages, neutrophils, and their interaction. Macrophages, owing to their plastic mode of activation, can generate a pro- or antitumoral microenvironment. Similarly, in autoimmune diseases, macrophages tip the Th1/Th2 balance via various effector cytokines. The contribution of neutrophils, an additional plastic innate immune cell population, to the microenvironment and disease progression is recently gaining more prominence in both cancer and autoimmune diseases, as they can secrete cytokines, chemokines, and reactive oxygen species (ROS), as well as acquire an enhanced ability to produce neutrophil extracellular traps (NETs) that are now considered important initiators of autoimmune diseases. Understanding the contribution of macrophages and neutrophils to the cancerous or autoimmune microenvironment, as well as the role their interaction and cooperation play, may help identify new targets and improve therapeutic strategies.

## 1. Introduction

The immune/inflammatory response is mostly beneficial to the host and is designed to combat and eradicate invading pathogens and then reestablish homeostasis. This universal response can also be activated in sterile inflammation, without any obvious infection, to repair excessive damage. The immune response is broadly categorized either as proinflammatory (consisting of Th1 and Th17 cells, M1-activated macrophages, and proinflammatory mediators designed to kill pathogens or tumor cells) or as anti-inflammatory (dominated by Th2 cells, M2-activated macrophages, and anti-inflammatory cytokines, designed to repair tissue damage). Of course, this approach is over simplistic, as more types of cell activation, including different types of regulatory T cells, macrophages, and B cells, are constantly being revealed.

In both cancer and autoimmune diseases an aberrant activation of the immune/inflammatory response leads to chronic diseases and accumulation of tissue damage. However, from an immunological standpoint, these two families of diseases are fundamentally different and even represent two opposite ways in which the immune system can go wrong. In cancer, the tumor cells are mostly unrecognized as antigens because a dominant anti-inflammatory response driven by the tumor cells suppresses any antitumoral immune response and promotes tumor progression and dissemination (immunosuppression). In fact, tumors are called wounds that do not heal, because the tumor hijacks the wound healing machinery and uses it to promote itself [1, 2]. In contrast, in autoimmune diseases, self-tolerance is broken and the inflammatory response is activated in excess against the host tissue cells, which express autoantigens that are misrecognized and attacked by the immune system, gradually leading to permanent tissue damage.

Differences between cancer and autoimmunity are evident even at the cellular levels. In solid cancers, the immune infiltrate is composed mostly of macrophages, as well as T regulatory cells (Tregs), some T effector cells (CD8 cytotoxic T cells), and NK cells, whereas other cell types, such as neutrophils, dendritic cells, and fibroblasts, remain mostly at the tumor rims. In contrast, autoimmune diseases are usually dominated by Th1 and Th17 cells and their cytokine products IL-2, IFN*γ*, and IL-17 (in Th1 autoimmune diseases such as rheumatoid arthritis, RA, multiple sclerosis, MS, and Hashimoto thyroiditis, HT) or by Th2 cells and their anti-inflammatory cytokines IL-4, TGF*β*, and IL-10 (in Th2 autoimmune diseases such as systemic lupus erythematosus, SLE, systemic or local sclerosis, SSc, or scleroderma). Relative to healthy individuals, Tregs are partially impaired in autoimmune patients, partly explaining the broken tolerance which characterizes autoimmunity [3, 4].

Multiple factors play a role in determining the outcome of the aberrant inflammatory process, including the type of inflicted tissue or organ, the degree of tissue injury sustained, the type of cells activated, the amounts of protein and lipid mediators that are locally and systemically secreted by those cells, and the extent to which immune regulatory checkpoints are activated. Collectively, these comprise the microenvironment.

Despite the many differences and the opposite activation of the inflammatory process as a whole, some interesting similarities exist between cancer and autoimmunity, particularly in the way phagocytes are activated and in shared processes like angiogenesis. In this review we attempt to highlight some similarities in microenvironmental elements between cancerous and autoimmune diseases, focusing specifically on the roles macrophages and neutrophils play in these diseases and how these similarities provide potential new avenues for their treatment.

## 2. A Causal Relationship between Cancer and Autoimmunity

In recent years the paradigm that chronic inflammation contributes to carcinogenesis has gained much support, but the reciprocal idea that cancer may invoke autoimmunity remains controversial. The fact that cancer and autoimmune diseases may sometimes occur in the same individual suggests a possible link between these two different clinical conditions. In such people, it is likely that the inflammatory process drives both autoimmunity and malignancy. However, it is unclear whether the autoimmune disease preexists and its chronic inflammatory process leads to malignancy in some of the cases (“inflammation-induced cancer”) or whether immune responses directed against tumor antigens eventually lead to autoimmune diseases (“tumor-induced autoimmunity”).

### 2.1. Can an Autoimmune Disease Cause Cancer?

Chronic inflammation has long been associated with increased risk of cancer. For example, patients with inflammatory bowel diseases (IBD, ulcerative colitis and Crohn's disease) have a 4–7-fold increased risk of developing colorectal cancer [13]. Autoimmune diseases are characterized as low-grade chronic inflammatory diseases that demonstrate leukocyte infiltration to the tissue, mostly by lymphocytes, and elevated levels of local and/or systemic inflammatory mediators, including cytokines, chemokines, and growth factors (e.g., IL-1*β*, TNF*α*, IL-6, CCL2/MCP-1, CXCL8/IL-8, and VEGF), reactive oxygen and nitrogen species (ROS, RNS), and autoantibodies [14]. The accumulation of these mediators results in slow and gradual tissue damage accompanied by somewhat increased angiogenesis and tissue remodeling, which is also called “smoldering inflammation” [13, 15]. This creates the “extrinsic pathway” linking inflammation and cancer [13]. Mechanisms that explain the extrinsic pathway include the generation of ROS/RNS that can cause DNA damage, the induction of the activation-induced cytidine deaminase (AID) by proinflammatory cytokines that results in accumulation of nucleotide alterations and increased genetic instability, and the role that key inflammatory transcription factors (e.g., NF-*κ*B and STAT3) play by inducing inflammatory cytokines and chemokines (e.g., IFN*γ*, IFN*α*, TNF*α*, and IL-17), as well as key cell cycle and survival proteins (e.g., Bcl2 family members, cyclin D, cIAPs, and c-FLIP) [reviewed in [13, 16, 17]]. Thus, chronic autoimmune diseases may indeed predispose patients to cancer over time. Many of these mediators (but not all) are products of phagocytes, especially neutrophils and macrophages, which affect tissue cells and drive their genetic instability.

### 2.2. Can Cancer Lead to an Autoimmune Disease?

An “intrinsic pathway” that takes place within tissue cells links cancer to inflammation, whereby genetic events that activate oncogenes or inhibit tumor suppressor genes may also lead to induction of inflammatory proteins. For example, EGFR activation may activate COX-2 through the activation of the transcription factors Sp1 and p38-mitogen-activated protein kinase (MAPK); the oncogene* ras* is involved in the induction of the chemokine IL-8/CXCL8; and PTEN mutations cause an upregulation of the transcription factor HIF-1, which, in turn, upregulates the chemokine receptor CXCR4 [summarized in [13]]. Mutated genes in tumors that elicit an immune response may also lead to initiation of an autoimmune disease; if the response is cross-reactive with the normal protein, the appropriate MHC haplotype is expressed, and the tissue specificity is correct. One example was found in patients with systemic lupus erythematosus (SLE) or Wegener's granulomatosis (WG) that were also diagnosed with cancer around the same time [18, 19], raising the question of which occurred first. A more detailed example is a group of scleroderma patients with increased risk of cancer that were shown to have developed autoantibodies to RNA polymerase III subunit (RPCI, encoded by the* POL3RA* locus), as opposed to other scleroderma patients with no cancer that had autoantibodies only to centromere B protein (CENTB) or topoisomerase-I [20, 21]. In these patients, both humoral and cellular specific immune responses were observed, suggesting that the mutations in the* POLR3A* gene, which are rare in human tumors, were the initiator event triggering an immune response.

## 3. The Microenvironment in Cancer and Autoimmunity

The microenvironment of inflamed tissues includes different cell types that secrete a myriad of mediators, including cytokines, chemokines, growth factors, lipid mediators, ROS and RNS, remodeling enzymes, and neuropeptides. These are derived from both tissue and stroma cells and orchestrate the recruitment of new cells into the inflammatory site, their interactions with each other, and their functions within the site. Although this occurs mostly locally within the tissue, these mediators may also exert systemic influences on remote organs, for example, at the premetastatic site in cancer or when autoimmunity spreads to several remote organs. Below, we discuss some aspects of the cancerous and autoimmune microenvironments that are common to both.

### 3.1. The Hypoxic Microenvironment

Low oxygen tensions (hypoxia) are observed in all inflamed tissues. Because different tissues exhibit a wide range of oxygen tensions, even under normal conditions, a functional definition determines that hypoxia results when the oxygen supply does not meet the oxygen demand of the cells [47]. Hypoxia stabilizes the hypoxia-inducible factors (HIFs), which are the master regulatory transcription factors that carry out the adaptation response of cells to hypoxia, including the shift to glycolysis, induction of angiogenesis, increased invasion of leukocytes, and immune suppression [reviewed in [48–52]]. Upregulation of HIF-1*α* induces angiogenesis and the shift to glycolysis, as HIF-1*α* binds to the hypoxia response element (HRE) found in the promoters of genes such as VEGF and the glycolytic enzymes. The switch to anaerobic glycolysis increases lactate levels, causing cellular acidosis and increased production of ROS, and leading to lipid peroxidation, membranal damage, impaired activity of ion channels, and increased membrane permeability. This increases spillage of cellular content and causes tissue acidosis and damage [52–54], which, in turn, recruit more leukocytes into the site and trigger inflammation. Hence, hypoxia and inflammation are interdependent, as chronic inflammation is accompanied by hypoxia and prolonged hypoxia leads to inflammation [55].

In cancer, the uncontrolled proliferation of tumor cells increases tumor mass, which becomes depleted of oxygen and nutrient supply as the tumor reaches a diameter of 2-3 mm, because of the increased distance from blood vessels. Since hypoxia is a major drive for angiogenesis, new blood vessels are produced to increase reoxygenation, and so different oxygen tensions can be measured in different regions within the tumor (Table 1) [56]. Partial pressure of oxygen values below 5 mmHg is measured in more than 50% of advanced solid tumors [57, 58]. Tumor cells are characterized by enhanced glycolysis, even in normoxic conditions (the Warburg effect), and hypoxia further enhances the anaerobic metabolism. The byproduct of glycolysis is lactic acid, which is transported out of the tumor cell to the microenvironment to prevent cell death by intracellular acidosis. Thus, neighboring stroma cells, particularly macrophages, are exposed to increased levels of lactate, which is actively transported into them. Lactate contributes to macrophage polarization by stabilizing HIF-1*α* and inducing expression of typical M2-phenotype markers like VEGF and arginase-I (ARG-I) [59], so that tumor-associated macrophages (TAMs) sense the metabolic changes in tumor cells and respond to them in a proangiogenic manner [1].

In autoimmune diseases, the increased infiltration of leukocytes into the inflamed site increases the demand for oxygen beyond the available supply. Low oxygen tensions were reported in organs with an ongoing inflammatory autoimmune process, such as the synovia in RA patients [10] and the pancreas in diabetes [60]. Thus, many macrophages infiltrate the synovium of RA patients, where they encounter a profound hypoxic microenvironment, upregulate HIF-1*α*, and mediate an angiogenic process that is necessary for the formation of the inflammatory pannus and leukocyte infiltration [51]. Likewise, migration of T cells and macrophages into the sclerotic lesions of MS patients generates a hypoxic microenvironment that drives secretion of proangiogenic factors, including VEGF, angiopoietins, and MMPs, and induces angiogenesis around the demyelinating plaques [61]. Increased serum lactate concentrations in MS patients correlate with disease activity score and reflect the hypoxic microenvironment [62]. The role of hypoxia and angiogenesis in diseases like systemic lupus erythematosus (SLE) is not clear, but it is known that about 50% of the patients suffer from anemia, which leads to tissue hypoxia and reduced oxygen delivery, especially, but not limited to, the pulmonary vascular beds [8]. Accordingly, elevated levels of proangiogenic factors, such as VEGF, FGF, PIGF, TNF*α*, TGF*β*, and HGF, were found in the serum of SLE patients [63]. Vascular disease, chronic tissue hypoxia, and excessive fibrosis that affects the skin and internal organs are the hallmarks of systemic sclerosis (SSc, scleroderma). An imbalance between proangiogenic factors (e.g., VEGF, PDGF) and antiangiogenic factors (e.g., angiostatin, thrombospondin-1) leads to increased serum levels of VEGF in early stages of the disease and increased serum levels of angiostatin in the late stage of the disease [64, 65].

Thus, in both diseases local hypoxia initiates a change in cell metabolism and elevates tissue acidosis, contributing to macrophage polarization and most importantly promoting the angiogenic switch, which is necessary for both cell survival and disease progression. Therefore, hypoxia and angiogenesis, although in different measures (Table 1), are two features of the microenvironment common to both cancer and autoimmunity.

### 3.2. Macrophages

Monocytes migrate into tissues and differentiate into macrophages that perform multiple, sometimes opposing functions that are needed in tissues, such as patrolling and maintaining homeostasis, eradicating tumor cells and pathogens, initiating wound healing and tissue repair, and resolving inflammation. These tasks are carried out by secreting inflammatory mediators (e.g., cytokines, chemokines, lipid mediators, and ROS/RNS) or anti-inflammatory mediators (e.g., IL-10, TGF*β*, and PGE_2_), presenting antigens to T cells and eliciting an adaptive immune response, scavenging apoptotic cells or necrotic debris, and depositing matrix proteins. Macrophages cannot perform all these tasks simultaneously, but they exhibit enormous plasticity, as they can be activated in different ways and constantly shift between them, according to the conditions in the changing microenvironment [66, 67]. This concept has been thoroughly reviewed before [68–74]. One extreme activation mode is the classically or M1-activated macrophages, which are activated to kill pathogens and tumor cells and accordingly express MHC class II and costimulatory molecules, Fc receptors to enhance phagocytosis, and proinflammatory and cytotoxic mediators (e.g., NO, TNF*α*). On the opposite extreme are the alternatively or M2-activated macrophages, which enhance wound healing and angiogenesis by expressing scavenger receptors (e.g., MARCO, CD206) and anti-inflammatory mediators (e.g., IL-10, TGF*β*, and PGE_2_), growth factors (e.g., VEGF), and matrix proteins. The hallmark of this type of activation is the high expression of arginase-I (ARG-I), which produces L-ornithine, the precursor for collagen synthesis, and polyamines that act as proliferative signals of cells. Another more refined approach further distinguishes between M2a, M2b, and M2c macrophages, where M2a are fibrotic, M2b are immune regulators and produce IL-1*β*, IL-6, and TNF*α*, and M2c are anti-inflammatory and are involved in tissue repair and remodeling [74]. In the continuum between the M1 and M2 options, macrophages can be activated in many forms of activation, which are very difficult to isolate and characterize. For example, regulatory macrophages are responsible for suppressing the Th1/M1 inflammatory response. Some of these cells are activated by Toll-like receptors (TLRs) ligands in combination with immune complexes, and some are activated by anti-inflammatory signals, such as adenosine or phagocytosed apoptotic cells [68, 72]. Immature monocytes/macrophages, which compose the monocytic myeloid-derived suppressor cells (M-MDSCs) population, also belong to regulatory macrophages and secrete IL-10 and TGF*β* to help suppress Th1 and CD8^+^ T cells and recruit regulatory T cells [75–77]. MDSCs inhibit T effector cells by expressing both inducible nitric oxide synthase (iNOS) and ARG-I that compete for their mutual substrate L-arginine, leading to its depletion and reduced production of CD3*ξ* chain in the TCR receptor, and therefore decreased antigen-specific T cell responses and proliferation [78].

In cancer, macrophages play a dual role. The concept of immunoediting [79] suggests that, in early stages of tumor development, the immune system successfully surveys and eradicates tumor cells. Tumor cells that survive remain constantly under immune pressure, which helps to “sculpt” their phenotype into a more aggressive one, until finally, at the third stage, they escape immune recognition and become established. This concept describes a close relationship between tumor and immune cells, which is crucial for the determination of tumor fate and progression [80]. Furthermore, it suggests that the regulation of the immune response is critical to the fate of the tumor: if the response is mostly proinflammatory, the immune cells will turn against the tumor and eradicate its cells, whereas if the response is anti-inflammatory, the immune cells will provide mediators that are necessary for tumor growth and promote tumor progression. Much progress has been made in recent years in our understanding of how tumor cells actively tip the balance and maintain a favorable, anti-inflammatory, and immunosuppressive response through their interactions with macrophages.

The majority of the macrophages found in the tumor originate from monocytes that were recruited to the site. Circulating monocytes are heterogeneous and are generally divided into at least two subsets: a major subset of classical monocytes (Ly6C^+^ in murine and CD14^++^CD16^−^ in human) and a minor subset of nonclassical monocytes (Ly6C^−^ in murine and CD14^+^CD16^+^ in human). There is currently controversy as to the role of different monocytes subsets in tumor progression. It has been suggested that nonclassical monocytes are preferentially recruited into the primary tumor, and classical monocytes are recruited more to the metastatic sites [81]. In contrast, other studies show that nonclassical patrolling monocytes have a role in preventing metastatic spread [82]. Furthermore, other methods of monocytes classification, based on different markers, are possible, although not yet common. For example, classifying monocytes according to their Tie-2 expression may be very relevant in cancer, as those monocytes are recruited into the tumor and have a profound and strong proangiogenic activity that is critical for tumor progression [83, 84]. This remains for now a subject of great interest.

Macrophages make up the major inflammatory cell population within tumors (Table 2), as they can infiltrate deep into the hypoxic microenvironment, unlike other leukocytes [85]. Several macrophage subsets have been found located in different regions of the tumor [86]. Tumor-associated macrophages (TAMs) are responsible for supporting tumor growth and dissemination. This is achieved by secreting IL-10 and TGF*β* which inhibit adaptive immune responses, VEGF, and other proangiogenic factors that promote angiogenesis, growth factors such as EGF that are necessary for the tumor cell viability, and matrix remodeling enzymes such as matrix metalloproteinases (MMPs) that enable cellular motility. TAMs are activated in a manner approximating M2-activation, and thus express ARG-I, produce matrix proteins, and secrete elevated levels of IL-10 and TGF*β*. Additional forms of macrophage activation in tumors include the Tie-expressing monocytes (TEMs), which are strongly proangiogenic and reside close to blood vessels [87] and MDSCs. MDSCs infiltrate the tumors and expand proportionally to the tumor burden [70, 72, 84]. TAMs, TEMs, and MDSCs are all obligatory components of the tumor microenvironment and share many similar markers and functions (especially TAMs and MDSCs), so it is very difficult to distinguish between them or to isolate them for* in vitro* studies.

Several microenvironmental conditions ensure that macrophages in tumors are activated in a way approximating M2-activation. First, the tumor cells secrete soluble mediators, such as M-CSF/CSF-1, VEGF, and TGF*β*, which recruit macrophages to the tumor and maintain their viability, while polarizing them towards M2-activation [88–90]. Second, the hypoxic microenvironment can shift even M1-activated macrophages towards M2-activation, utilizing multiple transcriptional and posttranscriptional mechanisms [48, 91–93]. Lastly, in a process called efferocytosis, macrophages engulf apoptotic cells, particularly apoptotic neutrophils (that were recruited to the tumor, secreted their content, and died by apoptosis; see Section 3.3), and this triggers M2-activation to promote angiogenesis, wound healing, and tissue remodeling [94]. Once M2 activated, these macrophages enhance their secretion of TGF*β* and IL-10, thus further immunosuppressing M1-activated macrophages in their vicinity. In contrast, macrophages that phagocytose tumor cells undergoing secondary necrosis, which release danger-associated molecular patterns (DAMPs) such as HMGB1, are M1-activated, lead to increased secretion of inflammatory cytokines (TNF*α*, IL-1*β*, and IL-12), and promote Th1 responses [95]. Thus, because of their plasticity, it is likely that, in the same tumor, some macrophages will be M1-triggered and most will be M2-activated, depending on their relative location within the tumor mass. This plasticity is now used in the treatment of cancer, as immunotherapy using monoclonal antibodies (e.g., anti-OX40, anti-EMMPRIN) was shown to modulate the microenvironment, reduce the levels of anti-inflammatory cytokines (e.g., TGF*β*), change the T cell infiltrate, and repolarize macrophages to become M1-activated, capable of killing tumor cells [96, 97]. Furthermore, drugs that alter TAMs activation were shown to enhance the effect of different immunotherapy approaches by changing the microenvironment [98]. However, the mechanisms that allow such manipulations are not entirely elucidated.

In contrast to cancer, the polarization state of macrophages in autoimmune diseases is poorly defined. Following the Th1/Th2 paradigm and extending it to the M1/M2 paradigm, one would expect to find M1 macrophages in Th1 autoimmune diseases such as RA, MS, and HT and M2 macrophages in Th2 autoimmune diseases such as SLE and scleroderma. However, the data is controversial. In one study, macrophages from the synovial fluid of RA patients expressed proinflammatory polarization markers (e.g., MMP12, CCR2), consistent with the elevated levels of proinflammatory cytokines detected in these patients' synovial fluids [123]. However, in another study, synovial fibroblasts were induced by TNF to secrete soluble factors that suppressed macrophage production of IFN*β* and limited macrophage ability to respond to IFN*β* by inhibiting Jak-STAT signaling, leading to decreased levels of M1-chemokines such as CXCL9 and CXCL10 [124]. In MS patients, activated microglia in preactive and remyelinating lesions expressed a mixed phenotype with both M1 markers (CD74, CD40, and CD86) and M2 markers (CCL22 and CD209, but not CD206) [125], whereas, in a mouse model of experimental autoimmune encephalitis (EAE), inhibition of the Aurora kinase blocked disease development and shifted macrophage phenotype from M1 to M2 [126]. In SLE, the contribution of macrophages to disease pathogenesis was hardly investigated. In a mouse model of SLE, generated by immunization with activated lymphocyte-derived DNA, macrophages infiltrating the nephritic tissues exhibited activation markers of M2b polarization (MHCII^high^CD86^+^IL-10^high^IL-12^low^) [127]. However, much evidence points to a possible mixed activation of macrophages in SLE, which includes both M1 and M2b polarized macrophages. For example, high levels of proinflammatory cytokines (e.g., TNF*α*, GM-CSF, IFN*γ*, CCL2, and CXCL10) are found in serum of SLE patients, alongside high levels of IL-10 and IL-6 [128]. Both systemic and localized sclerosis (scleroderma) are autoimmune diseases manifested by vascular injury and progressive fibrosis of the skin, lung, and internal organs. The cytokine balance in these conditions is shifted towards Th2 cytokines, such as TGF*β*, PDGF, IL-4, and IL-13. Accordingly, macrophages are M2-polarized with high expression of the CD206 marker [129]. Interestingly, this shift towards M2 was shown to be mediated by the enzyme N-acetylglucosaminyltransferase-V (GnT-V) that glycosylates surface proteins, as mice with deficiency in the gene (MGAT5^−/−^) were resistant to bleomycin-induced scleroderma and showed decreased M2-activation of cutaneous macrophages, with a similar total count of macrophages as the wild type mice [130].

The role of macrophages in cancer diseases has been investigated in depth, whereas their role in autoimmune diseases merits more research. The plasticity of macrophages and their ability to respond to changing conditions suggest that their polarization* in vivo* is difficult to assess. Unlike the defined* in vitro* stimulus, mixed signals in the complex microenvironment* in vivo* may result in different subpopulations of macrophages exhibiting different polarization and different functions. It is, therefore, important to precisely define the conditions in the microenvironment in each disease and to understand how these change over time in different parts of a tumor, in different organs, and in different stages of disease development. Furthermore, the mixed polarization of macrophages that is observed* in vivo* can be the result of intermediate transitioning from one polarization to another, or a result of a complex tissue structure that includes niches or even microniches that exhibit small nuances in the microenvironment. It is also important to remember that although most macrophages are recruited from the circulation during inflammation, some macrophages are resident in the tissue. At present, the specific role of tissue resident macrophages within the tumoral or autoimmune microenvironment is not well understood, mostly because of our current inability to distinguish them from recruited monocytes and due to their scarcity within the microenvironment. This is further complicated by the fact that, in some tissues, such as the intestine and heart, resident macrophages are gradually replaced by monocyte-derived macrophages [131, 132], whereas, in the brain, resident microglia are long-lived and can proliferate to maintain their numbers independently of monocyte infiltration [133]. The question whether these resident macrophages have different roles than the infiltrating monocyte-derive macrophages remains unresolved, but at least, in the murine model of EAE, microglia seem to be activated in early stages of disease development, supporting this premise [133]. Lastly, a new field of study of the trained innate immunity now demonstrates how innate immune cells may acquire a memory through epigenetic reprograming [134]. The significance of this subject to the activation of macrophages awaits further investigation and raises the question of how the history of the macrophages affects their ability to respond to the changing microenvironment and polarize correctly.

### 3.3. Neutrophils

Neutrophils were viewed as cells that terminally differentiate in the circulation, migrate into tissue in response to inflammatory signals, degranulate in response to triggering, and die of apoptosis immediately after. However, recent findings challenge this concept and place neutrophils, together with macrophages, as cells that secrete a myriad of regulatory mediators that shape their immediate microenvironment, all depending on the diverse cell types they meet.

In cancer, and using an analogy to the M1- and M2-activation modes of macrophages, neutrophils are now also categorized as antitumoral N1 and protumoral N2 tumor-associated neutrophils (TANs) [33]. Neutrophils make up a relatively small percentage of the tumor mass and are primarily found at the tumor rims and in nonnecrotic areas. They can infiltrate the tumor in small numbers (Table 1) and then are often found near blood vessels or in compact aggregations. However, changing the tumor microenvironment by blocking TGF*β* signaling increases neutrophil infiltration and reduces tumor size [33]. TANs within the primary tumor are protumoral, as they secrete the proangiogenic factor Bv8, which is also responsible for myeloid cells recruitment, especially at early stages of malignancy [135], as well as the proangiogenic matrix metalloproteinase MMP-9, both in larger amounts than their cognate TAMs [30]. Furthermore, once TGF*β* is blocked, a collaboration between TAMs and TANs is demonstrated, as TAMs produce neutrophil chemoattractants that recruit CD11b^+^Ly6G^+^ neutrophils into the tumors [33]. Note that mature neutrophils and immature granulocytic MDSCs are practically indistinguishable, as they both express the same surface markers (CD11b^+^Ly6G^+^), and it is yet unclear whether mature neutrophils arrive at the tumor from the circulation or whether immature MDSCs mature to N2 TANs within the tumor [33].

Neutrophils make up a much smaller fraction of the immune infiltrate in the tumors compared to macrophages, but their relative contribution is still unclear. For example, in some tumors, they may be the main producers of MMP-9 and not the more abundant macrophages [30]. It is clear that the contribution of CD11b^+^Gr1^+^ granulocytic MDSCs to the formation of the premetastatic niche is significant. These granulocytic MDSCs (and not monocytic MDSCs) infiltrate the lung premetastatic niche well before tumor cells arrive there and secrete* in situ* large amounts of MMP-9, resulting in aberrant and leaky vasculature in the premetastatic lung. In addition, these G-MDSCs inhibit the secretion of IFN*γ* by lung macrophages and increase the secretion of anti-inflammatory cytokines, such as IL-4, IL-5, and IL-10, indicating an immune suppression of the lung [136]. They can also secrete the neutrophil chemoattractants S100A9 and S100A8, as well as the proangiogenic Bv8. Interestingly, Bv8 also induces the migration of metastatic cells, suggesting that G-MDSCs direct the homing of metastatic tumor cells into the lung [137]. In contrast, other studies demonstrate that depletion of Ly6G^+^ neutrophils does not change the size of the primary tumor but increases the lung metastatic burden, suggesting that tumor entrained neutrophils (TENs) at the premetastatic niche inhibit, rather than promote, metastasis [138]. Furthermore, adoptive transfer of such TENs significantly inhibited formation of lung metastatic foci, as they were highly cytotoxic to tumor cells. This cytotoxicity is triggered by the tumoral secretion of CCL2. However, neutrophils become cytotoxic only at the premetastatic lung and not at the primary tumor site where they are subjected to high levels of the local inhibitory effects of TGF*β* [138]. This antitumoral effect of TENs was only temporary, and eventually they failed to inhibit metastasis. Thus, neutrophils display different functions at the primary tumor site and at the premetastatic niche, and within the metastatic niche their role changes over time.

Unlike macrophages, neutrophils can produce the proinflammatory cytokine IL-17, to mediate their involvement in cancer. IL-17 is mainly produced by either neutrophils or Th17 lymphocytic cells. High IL-17 levels or high frequency of cells producing IL-17 correlates with poor prognosis, whereas high Th17 cell frequencies were correlated with improved prognosis [139], suggesting that neutrophils might be the culprits. In fact, in different tumor types (e.g., head and neck, ovarian, endometrial, prostate, breast, lung, and colon carcinomas), IL-17 was mostly produced by neutrophils (66% of the IL-17 producing cells in the tumor mass), whereas Th17 cells constituted only a small fraction of the immune infiltrate producing IL-17 (4%) [140]. In contrast, other studies suggested that IL-17 was secreted by Th17 or *γδ* T cells, which were responsible for neutrophil recruitment into the tumors. The recruited neutrophils, in turn, immunosuppressed CD8^+^ cytotoxic T cells and promoted angiogenesis and metastasis [141, 142]. Thus, although IL-17 is considered proinflammatory, its correlation with poor prognosis suggests that it also has protumoral roles. For example, IL-17 can increase tumor cell growth and migration [140, 141], induce IL-6 and CCL20 that recruit Th17 to the tumor site, and modulate gene expression of nontumor cells (including enhanced production of cytokines and chemokines, transcription factors, and antiapoptotic proteins), suggesting that neutrophils play an important role in tumors at an early stage [141].

In some autoimmune diseases, neutrophils are a major component of the immune infiltrate. For example, in RA, 90% of all leukocytes in the joint may be neutrophils [143], suggesting that they have a significant contribution to the pathogenesis of the disease. Although generally the role of neutrophils in autoimmune diseases has not been thoroughly investigated, their importance is now gradually gaining acceptance. Several possible mechanisms of action for neutrophils in autoimmune diseases have been suggested, as follows.

Neutrophils are phagocytes with a strong cytotoxic potential, and when activated in a proinflammatory manner (N1) they can enhance their secretion of proteases and ROS and, in an autoimmune context, inflict tissue damage. They also secrete chemokines that attract more neutrophils, macrophages, and other stroma cells into the inflamed site, thus amplifying the destructive effect in this context. In RA, migration of neutrophils to the joint is regulated by their enhanced expression of chemokine receptors (e.g., CCR2) that lead them towards elevated levels of CCL2 found in the synovial fluid (SF) [144]. Furthermore, IL-17 that is produced by neutrophils is an important mediator in arthritis, as IL-17 KO mice exhibit a clinical score less severe than wild type mice in the K/BxN serum-induced arthritis model [145]. In MS patients, circulating neutrophils are primed compared to healthy controls and exhibit reduced apoptosis and enhanced expression of surface markers (e.g., TLR2, IL-8 receptor) [146]. Disruption of the blood brain barrier (BBB) in MS patients or in mice with EAE allows entry of leukocytes, including neutrophils, into the brain. Secretion of IL-17 by both Th17 and neutrophils helps to further disrupt the BBB and attract even more neutrophils and macrophages to the site of inflammation, especially at the preclinical stage before disease onset [43]. In patients with type I diabetes (T1D), the role of neutrophils remains controversial; however several observations indicate that circulating neutrophils are slightly reduced during the early stages of the disease and that they are accumulating at the exocrine pancreas in very small blood vessels or adjacent to acinar cells [147]. Neutrophils that are triggered by immune complexes are found in SF of RA patients, along with elevated levels of ROS [143]. In fact, neutrophils carrying the R620W polymorphism in the tyrosine phosphatase* Lyp*, which is highly expressed in neutrophils, exhibit enhanced migration and extravasation through endothelial cells, increased Ca^2+^ influx, and increased ROS production upon stimulation [148], demonstrating the importance of this polymorphism in the susceptibility to autoimmune diseases.

In another possible mechanism of action in autoimmune diseases, neutrophils have the ability to produce the enzyme peptidyl arginase deaminase-4 (PAD-4), which modifies the amino acid L-arginine into L-citrulline and is therefore involved in the generation of autoantibodies against citrullinated proteins found in both RA and MS patients in early stages [149, 150]. Moreover, neutrophils can release chromatin extracellular traps (neutrophil extracellular traps, NETs) in a process termed “NETosis” (or “ETosis” when other cell types, such as mast cells, eosinophils, or macrophages, perform it, although less efficiently). These NETs are composed of chromatin fibrils, a combination of DNA and proteins, including histones (70% of the proteins), HMGB1, neutrophil elastase (NE), myeloperoxidase (MPO), the peptide LL-37, and the hCAP18 fragment of cathelicidin. These proteins are recognized by immune cells (e.g., dendritic cells) as alarmins or danger-associated molecular pattern (DAMP) molecules when they are bound to DNA and spilled out of the cells. Citrulline is uncharged in neutral pH, as opposed to arginine, and can change protein folding, structure, and function. Some proteins may naturally include citrulline (e.g., myelin basic protein, MBP, several histone proteins), whereas others undergo citrullination in the inflammatory site (e.g., fibrin and fibrinogen in RA joints). When these proteins are posttranslationally modified by citrullination, neoepitopes may be revealed that are no longer tolerated, leading to the production of proinflammatory cytokines such as TNF*α*, IL-6, and IFN*α* [reviewed in [151]]. Low density granulocytes (LDG), a subset of immature neutrophils whose numbers increase in SLE patients, are particularly susceptible to NETosis, as they secrete IFN*α* [152]. NETosis is associated with the finding of antineutrophil cytoplasmic antibodies (ANCA) found in many SLE patients [153, 154] and is consistent with the finding of IFN*α* in the pancreas of T1D patients and the finding of IFN*α* and NETs in nonobese diabetic (NOD) mice that spontaneously develop T1D [reviewed in [147]]. In MS patients, higher serum levels of NETs were found [146].

Thus, neutrophils secrete many proinflammatory and cytotoxic mediators leading to the aggravation of the inflammatory response and culminating in gradually accumulating tissue damage, and they can cast NETs that lead to generation of autoantibodies, thus providing a hint to the etiology of autoimmune diseases. Both these mechanisms highlight neutrophils as significant and important cells in the generation of autoimmune diseases.

Neutrophils clearly play a large role in the microenvironment of both cancer and autoimmunity, but they are not as well understood as their “sibling” macrophages. Evidence suggests that they play a crucial role during early stages of diseases, but their role in later stages requires more investigation.

### 3.4. Macrophage-Neutrophil Cooperation

Macrophages and neutrophils show a high degree of overlap or redundancy as they secrete similar mediators, such as ROS, MMPs, cytokines, and chemokines. However, there are differences in the quantities produced and in gene expression. For example, both cell types secrete MMP-9 but in different quantities, and neutrophils, but not macrophages, can also secrete MMP-8; both phagocytes produce ROS, but neutrophils produce more hypochlorous acid; macrophages are by far better antigen presenting cells, whereas neutrophils excel in casting NETs. Both cell types are of myeloid origin and, therefore, have similar surface markers. Both types of cells exhibit similar plasticity, where the M1/N1 activation is geared to perform killing functions, whereas M2/N2 activation is directed towards healing wounds and promoting angiogenesis.

There is now some evidence of cooperation between macrophages and neutrophils. Both cell types secrete cytokines and chemokines that recruit each other and enhance each other's proinflammatory activities, thus enhancing resolution of inflammation [155] (see Table 3 for details of some cytokines and chemokines in the microenvironment). Macrophages secrete the macrophage migration inhibitory factor (MIF) to enhance neutrophil survival and secretion of MMP-9, in the context of both cancer [156] and autoimmunity [157]. The manner by which neutrophils die profoundly affects macrophage polarization, and, therefore, the subsequent course of disease. In cancer, in the absence of activating signals, neutrophils have a short half-life of 6–18 hours in the circulation, before dying by apoptosis, and the process of their engulfment and processing by macrophages (efferocytosis) results in macrophage polarization towards M2-like activation and enhances immunosuppression [158]. Furthermore, neutrophils secretion of IL-17 helps to shift macrophage activation towards the M2b regulatory phenotype [159]. In contrast, in autoimmune diseases, presence of GM-CSF and hypoxia can delay neutrophil apoptosis and increase their survival [143]. Moreover, in early RA patients, antiapoptotic cytokines (e.g., IL-4, GM-CSF, and G-CSF) that are found in their SF may lead to defects and low levels of apoptotic death in neutrophils, suggesting that their engulfment by macrophages after secondary necrosis elicits a proinflammatory response.

This evidence suggests that macrophages and neutrophils communicate with each other and cooperate to regulate the microenvironment, explaining why both cell types seem to play similar roles in clinical settings. It has even been shown that when macrophages are depleted, or even change their activation mode, neutrophils gain the ability to infiltrate a tumor instead [98]. Therefore, myeloid cells and molecules that mediate their cooperation become new attractive targets for cancer immunotherapy. However, many questions that merit further investigation remain unanswered. For example, what factor(s) direct the tumor microenvironment, so that M2-TAMs become the dominant cellular component, rather than N2 TANs? Can macrophages compensate for the lack of neutrophils, or are neutrophils necessary for tumor growth, despite their being such a small percentage of the tumor mass? To what extent is this cooperation between macrophages and neutrophils necessary for tumor progression or for the development of the metastatic niche? And finally, is there a direct interaction between these two cell types, and if so, what protein(s) mediate it? Similarly, various interesting possibilities exist for the study of the role macrophages-neutrophils interactions play in autoimmune diseases.

### 3.5. Autoantibodies

Antibodies are effector molecules that specifically bind to their antigens and thus tag the cell for destruction either via complement fixation or via other effector cells (e.g., macrophages, NK cells) that have the appropriate Fc receptor. The binding of antibodies can also promote or inhibit cell signaling and activation. During early stages of an autoimmune disease, the process of NETosis exposes many citrullinated self-proteins to the immune system, and since the modification renders these proteins neoantigens, tolerance is broken and the immune system can generate autoantibodies and enhance epitope spreading, resulting in autoimmune responses [149, 151]. Other posttranslational modifications (PTM), such as carbamylation and oxidation, can also generate neoantigens and autoantibodies [160]. The binding of these autoantibodies to their modified targets may drive tissue damage through their effector functions and contribute to the generation of autoimmune diseases [161], suggesting a causative role for the autoantibodies. However, it should be remembered that NETosis is a physiological and protective process (e.g., limiting invading pathogens) that does not necessarily lead to an autoimmune response. Additional factors (e.g., specific genetic background of an individual, specific polymorphism in genes related to NETosis, and defects in the mechanisms responsible for the clearance of NETs) must also exist to allow an autoimmune disease to develop [154].

In many autoimmune diseases autoantibodies can be found in the serum of patients and these may have critical role in the pathogenesis of these diseases through aberrant signaling of cells or through their destruction. In fact, autoantibodies can be considered a hallmark of autoimmune diseases and are therefore often used as biomarkers for disease progression. For example, presence of autoantibodies against insulin, GAD65, and IA-2 can confirm the diagnosis of type I diabetes (T1D) [147], and anti-dsDNA antibodies bind to resident kidney cells and trigger signaling that promotes inflammation and fibrosis in SLE [162]. Antinuclear antibodies (ANAs) are widely used as diagnostic biomarkers, and they have been shown to be involved in the pathogenesis of several autoimmune diseases, particularly systemic autoimmune diseases, as they form immune complexes with their target proteins and generate inflammation in many organs, like the kidney, lung, skin, brain, joints, and others [163]. Some ANAs are associated with specific diseases. For example, autoantibodies to double-stranded DNA and antihistones are associated with SLE, whereas anti-DNA-topoisomerase-I and anti-centromere protein B (CENTB) are linked to scleroderma [163].

Autoantibodies can be found in patients with inflammatory diseases that may ultimately progress into cancer, such as chronic hepatitis and liver cirrhosis, even in early, precancerous stages. Once cancer progresses, many autoantibodies can be found in different types of solid cancers, directed against over 100 tumor-associated antigens (TAAs), including autoantibodies to CA-125, chromogranin A, and plasminogen [164–166]. However, some of these autoantibodies overlap with autoantibodies found in patients with autoimmune diseases, such as different ANAs (e.g., anti-Sm, anti-CENTB), autoantibodies to double-stranded DNA, p53, and c-Myc [167, 168]. Autoantibodies to citrullinated proteins were found significantly more frequently in the sera of diffuse large B-cell non-Hodgkin lymphoma patients than in healthy controls [169]. The presence of such autoantibodies in cancer may be explained by the increased necrotic death of tumor cells, combined with neutrophil-derived NETosis and proteolysis of spilled proteins that may reveal cryptic epitopes. However, the role these autoantibodies play in cancer is still undetermined. It is possible that such autoantibodies may confer partial protection from cancer by promoting tumor cell death through complement-dependent cytotoxicity (CDC) or macrophage-mediated antibody-mediated cell cytotoxicity (ADCC), at least in early stages of cancer development. This has been shown for anti-TPO and anti-Tg autoantibodies in patients with both Hashimoto's thyroiditis and papillary thyroid cancer [14]. Other protective effects, such as inhibition of protein activity or induction of cell cycle arrest, should also be investigated. However, it is likely that, in later stages of tumor growth, the immunosuppressive microenvironment hampers those effects. Clearly, the relevance of autoantibodies to tumor pathogenesis merits more investigation.

Antibodies are, therefore, components in the microenvironment of both autoimmune and cancerous diseases. Although they are known to be very powerful effector molecules, the pathogenic role of antibodies in these diseases, especially in cancer, remains not fully elucidated, and it is possible that lessons learnt in one clinical scenario will improve our understanding of the other.

## 4. Concluding Remarks

We reviewed here several aspects of the microenvironment in two clinically and immunologically opposing diseases and showed that, despite their fundamental differences, there are some instructive parallels between them. For example, hypoxia and angiogenesis are a common denominator in both diseases, although oxygen tensions may be variable and not comparable* per se*. Likewise, the presence of autoantibodies is a similar feature, especially when autoantibodies against the same self-antigens are involved. In this respect, it is likely that research of these elements in the context of one disease will shed light on their role in a different disease.

Innate immunity, and specifically myeloid cells, has long been recognized as crucial for tumor progression and metastasis, whereas its role in autoimmune diseases is only beginning to be unfolded. The paradigm that autoimmune diseases are mediated exclusively by B and T cells of adaptive immunity is gradually shifting to one recognizing the vital role that myeloid cells play as drivers and regulators of the microenvironment and of autoimmune responses. The adaptive immune cells (T and B lymphocytes) must be activated by antigen presenting cells, a process requiring the prolonged activation of both macrophages and neutrophils. In particular, after macrophages were recognized as cells with enormous plasticity that respond to and regulate a changing microenvironment, this concept has extended to recognize similar properties in neutrophils in both cancer and autoimmune diseases. In view of the chronicity of both cancer and autoimmune diseases, the paradigm that neutrophils are short-lived and fully differentiated cells now shifts to include the understanding that neutrophils can extend their survival according to conditions in the microenvironment. Indeed, the newly discovered involvement of neutrophils in both cancer and autoimmunity and the importance of the interactions between neutrophils and macrophages present a novel field of study, which will probably expand in the future.

Lastly, identifying the parallels in these two clinically opposing diseases may provide us with new targets and tools for therapy. For example, the ability of macrophages to home in on the hypoxic regions in tumors leads us to use these cells as vehicles to deliver gene therapy [170]. Amazing progress has been made in immunotherapy during the last few years, where different regulatory checkpoints and “go signals” are targeted in an attempt to change the microenvironment. In autoimmune diseases such as RA, anti-TNF biologics are now routinely administered and improve life quality for many patients, and, in cancer, we have recently witnessed the success of combined anti-CTLA4 and anti-PD-L1/PD-1 in the treatment of melanoma [171]. Targeting the process of leukocytes recruitment into inflamed sites is now gaining more success. Using CCR2 antagonists inhibited tumor growth and prevents metastasis [81, 172], as well as reducing inflammation and joint destruction in a murine model of adjuvant-induced arthritis [173]. Additional targets, such as the CSF-1 receptor kinase or CX3CL1, lead to macrophage depletion and greatly improved kidney pathologies in mouse models of nephritic lupus [174, 175]. Neutrophil recruitment can also be targeted by blocking CXCL8 or CXCL6 signaling with antibodies, and this approach has produced similar benefits in inhibiting tumor growth and metastasis [176, 177]. Other strategies that target the immunosuppressive microenvironment, specifically by targeting different steps in TGF*β* signaling pathway, also show efficacy in reducing invasiveness, migration, and tumor size in murine models of breast [178, 179], glioma [180], and colon cancer [181]. This targeting of TGF*β* pathway ameliorated immunosuppression and shifted the cellular composition within tumor microenvironment towards increased CD8^+^ T cells, macrophages, and NK cells [180].

These novel and promising immunotherapies can be further extended with novel targets, like anti-IL-6 receptor, anti-CD20, and many others that are already in the pipeline. By studying the parallels and differences between cancer and autoimmunity, other potential targets could be identified and appropriate strategies developed to achieve the desired outcome of treatment for cancer and autoimmune diseases.

## Figures and Tables

**Table 1:** Hypoxia in the microenvironment.

	Cancer tissue	RA (SF)	MS/EAE	SLE
Hypoxia	≤5 mmHg (70–80 *μ*m from vessel); ≤0.5 mmHg (≥150 *μ*m from vessel); [5] 0–2.5 mmHg (breast cancer) [6]	18–24 mmHg	9–20 mmHg in EAE [7]	Not directly measured; anemic hypoxia reported [8]

Normal tissue	Range: 25–72 mmHg (depending on tissue) [9]	40–70 mmHg [10–12]	35 mmHg [7]	

**Table 2:** Examples for the distribution of macrophages and neutrophils in different types of cancer and autoimmune diseases.

Type of carcinoma	Localization	Percentage (%)	Mice/human	Ref
*Macrophages in cancer*				
Mammary gland Gastrointestinal tumors Diffuse large B-cell lymphoma Non-small-cell lung cancer (NSCLC)	Macrophages are found infiltrating all areas of the tumors (including the perinecroptic areas) Many macrophages are found in the stroma, in close contact with the cancer cells	>40% <30% 20% 15%–30%	Mice Human Human Human	[22] [23] [24] [25, 26]
Prostate cancer	In stroma and in close contact with cancer cells	10%–15%	Human	[27]
Pancreatic cancer	Intratumoral and in the invasive front	30–50%	Human	[28]
Colon cancer	Intratumoral, numbers increase with tumor stage and grade	25%–50%	Human	[29]
L929 Fibrosarcoma, B16 melanoma, LLC lung carcinoma cells	Intratumoral	23–51%	Mice	[30]

*Neutrophils in cancer*				
Lung cancer	Infiltrating the tumor	8%	Human	[31]
Clear cell renal cell carcinoma (RCC)	Intratumoral or near vessels	14%	Human	[32]
Mesothelioma, lung cancer	Intratumoral	0.7–2.5%	Mice	[33]
L929 Fibrosarcoma, B16 melanoma, LLC lung carcinoma cells	Intratumoral or near vessels	3–8%	Mice	[30]

*Macrophages in autoimmune diseases*				
Rheumatoid arthritis (RA)	Lining the synovial membrane	41%	Human	[34]
	Lining and infiltrating the synovium	35–46%	Human	[35]
	Lining and infiltrating the synovium	17–36%	Human	[36]
	Infiltrating the synovium	26%	Human	[37]
Multiple sclerosis (MS)	Infiltrating and at the rim of the lesion	15–30%	Human	[38]
Systemic lupus (SLE)	Kidney: infiltrating all parenchyma, found surrounding glomeruli and around perivascular aggregate	26%	Mice	[39]
Systemic lupus (SLE)	Throughout the nephritic kidney	4%	Mice	[40]
Scleroderma	Skin	23%	Rat	[41]
Systemic sclerosis (SSc)	Superficial and deep dermis at early stages	13%	Human	[42]

*Neutrophils in autoimmune diseases*				
Rheumatoid arthritis (RA)	Lining and infiltrating the synovium	8–15%	Human	[35]
	Infiltrating the synovium	4.5–7%	Human	[37]
Experimental autoimmune encephalomyelitis (EAE)	Within brain lesions In the spinal cord	0.4–3% 8%	Mice	[43–45]
Systemic lupus (SLE, juvenile)	CD15^+^ low density granulocytes in circulation	10%	Human	[46]

**Table 3:** Example concentrations of cytokines and chemokines in the microenvironment.

		Cancer (breast, pg/mL/mg)^a^	RA (SF^b^, pg/mL)	MS/EAE (CSF^c^, pg/mL)	SLE (serum, pg/mL)
IL-1*β*	Disease	2.7–3.5 [99, 100]	2.6 [36] 9.26 [101]	0.02 [102] 44.1 [103]	0.24 [104] 11 [105]
	Healthy/remission	0 [100]	0 [36] 7.7 [101]	0 [102]	0.1 [104] 5 [105]

TNF*α*	Disease	7.2 [100]	14.0 [106]	1.85 [102] 5.34 [107] 9.0 [108] 39.4 [103]	0.34 [104] 1.24 [109] 7.8–8.0 [110, 111] 44.76 [105]
	Healthy/remission	1.6 [100]	3.5 [106]	0.93 [102] 1.95 [107]	0.1–2.2 [104, 109, 111] 20 [105]

IFN*γ*	Disease	27.6 [100]	0 [36]	3.27 [102] 5.7 [107] 11.6 [108]	0.64 [104] 6.5–7.05 [109, 110]
	Healthy/remission	16.6 [100]	0–3.5 [36, 106]	0.2–0.52 [102, 108] 3.7 [107]	1.3–11.7 [104, 109, 110]

IL-17A	Disease	0 [100]	0 [36] 12 [112]	6.93 [102] 16.53 [107]	97.42 [109]
	Healthy/remission	0 [100]	0 [36] 4 [112]	3.36 [102] 13.7 [107]	3.30 [109]

IL-6	Disease	17.2 [100]	1,253 [36] 355 [101]	2.86 [102] 6.02 [107] 13.2 [103, 108]	10.02 [109] 20.8 [110] 70.45 [105]
	Healthy/remission	1.2 [100]	1,170 [36] 87 [101]	2.5–12 [102, 108] 6.24 [107]	0.5–2.18 [109, 110] 20 [105]

TGF*β*	Disease	86.7 [113]	768 [36]	74.6 [107]	42,990 [109]
	Healthy/remission		0 [36]	64 [107]	82,710 [109]

IL-10	Disease	0.3 [100]	16.2 [36]	0.95 [102] 4.34 [107]	1.2 [111] 2.82 [31, 104] 9.78 [109]
	Healthy/remission	0 [100]	0 [36]	0–0.63 1.13 [102] 0.38 [107]	0.54 [104, 109, 111]

CCL2/MCP-1	Disease	121 [100]	25,000 [114]	116.3 [108] 574.4	136 [115]
	Healthy/remission	1.9 [100]	920–2900 [114]	163–526 [108, 116]	71 [115]

VEGF	Disease	1,148 [117]	1,100 [118] 1,800 [119]	Below the level of detection [120]	300.8 [121]
	Healthy/remission	163 [117]	700 [119]	Below the level of detection [120]	124 [121]

IL-4	Disease	1.7–3.1 [99, 100]	0 [36]	0.17 [102] 3.3 [107] 8.6 [116]	0.1–0.2 [104, 110]
	Healthy/remission	0 [100]	0 [36]	0.03–0.1 [102, 116] 1.74 [107]	0 1–0.3 [104, 110]

IL-8	Disease	68 [100]	584 [101]	30–35 [102, 122]	358 [111]
	Healthy/remission	1 [100]	451 [101]	28–31 [102, 122]	150 [111]

IL-12	Disease	2.3 [100]	10.5 [106]	1.44 [102] 4.9 [116]	1.0 [104]
	Healthy/remission	1.4 [100]	6.1 [106]	0.56–1.4 [102, 116]	0.18 [104]

^a^Measured in tumor extracts.

^b^Measured in the synovial fluid (SF).

^c^Measured in the cerebrospinal fluid (CSF).
